# Isolated Superficially Serrated Adenoma in Ulcerative Colitis: A Case Report with Molecular Analysis in Japan

**DOI:** 10.3390/diagnostics16193147

**Published:** 2026-09-28

**Authors:** Mitsuhiro Tachibana, Sayaka Choki, Ryo Sugimoto, Masahiro Matsushita, Tamotsu Sugai

**Affiliations:** 1 Department of Diagnostic Pathology, Shimada General Medical Center, Shimada 427-8502, Shizuoka, Japan; 2 Department of Gastroenterology, Shimada General Medical Center, Shimada 427-8502, Shizuoka, Japan; 3 Department of Gastroenterology, Ebina General Hospital, Ebina 243-0432, Kanagawa, Japan; 4 Department of Molecular Diagnostic Pathology, School of Medicine, Iwate Medical University, Shiwa 028-3695, Iwate, Japan; 5 Department of Diagnostic Pathology, Southern Tohoku General Hospital, Kōriyama 963-8052, Fukushima, Japan

**Keywords:** rectum, superficially serrated adenoma, KRAS, RSPO, ulcerative colitis

## Abstract

**Background and Clinical Significance:** Superficially serrated adenoma (SuSA) is a recently recognized colorectal serrated lesion characterized by superficial serration and adenoma-like deep glands. Its clinicopathological significance in ulcerative colitis (UC) remains unclear. **Case Presentation:** We report a rectal SuSA in a 57-year-old Japanese woman with a 30-month history of UC and a history of follicular lymphoma. UC was in a mild, resolving phase at the time of detection. A 7 mm pale, sessile rectal lesion was completely removed by cold snare polypectomy. Histopathologically, the lesion showed superficial serration confined to the upper one-third to one-half of the glands, adenoma-like deep glands, and a basal-to-middle proliferative zone. No ectopic crypt formation, basal crypt dilation, L-/T-shaped crypt architecture, or adjacent dysplasia was identified. Molecular analysis revealed a *KRAS* p.Q61H mutation and *PTPRK::RSPO3* fusion retaining the *RSPO3* coding region. The morphological and molecular features were consistent with sporadic SuSA rather than inflammatory bowel disease-associated non-conventional serrated dysplasia. The short duration of UC and mild, resolving inflammation did not support a causal relationship between UC and SuSA development. To our knowledge, this is the first genetically characterized case of SuSA in a patient with UC in Japan. **Conclusions:** This case expands the limited molecular data on SuSA arising in UC. Whether SuSA occurs more frequently in patients with UC than in the general population remains unknown and will require population-based and multicenter clinicopathological and molecular studies.

## 1. Introduction

Superficially serrated adenoma (SuSA) was proposed by Japanese pathologist T. Hashimoto and colleagues in 2018 as a new type of serrated lesion characterized by distinct histological features [1]. Histopathologically, SuSA exhibits features of both conventional-type tubular adenomas and serrated lesions, making classification challenging. Notably, the serrated neoplasia pathway accounts for approximately 30% of cases of sporadic colorectal cancer [2].

Because SuSA is a precursor lesion of the *KRAS*-subtype traditional serrated adenoma (TSA), it may have malignant potential [3,4,5,6]. Ulcerative colitis (UC) carries an increased risk of colorectal cancer due to the chronic nature of the disease, widespread inflammation, and persistent inflammatory stimulation [7]. SuSA frequently exhibits concurrent *KRAS* mutations and *RSPO* fusion or overexpression (Table 1). *KRAS* primarily activates MAPK signaling, whereas *RSPO* alterations are associated with WNT pathway activation and are common to SuSA and TSA [1,8,9].

Serrated lesions, including sessile serrated lesions (SSLs) and TSA-like lesions, have also been described in patients with inflammatory bowel disease (IBD). However, their clinicopathological significance and relationship to IBD-associated dysplasia remain incompletely understood [10,11,12,13]. To date, Gao et al. in China reported only one case of SuSA in a patient with UC in 2025 [14]. They did not perform genetic analysis of the tumor.

Here, we report the first genetically characterized Japanese case of SuSA associated with UC in Japan and compare it with previously reported cases of SuSA without associated background lesions.

**Table 1:** Clinicopathological and molecular features of superficially serrated adenomas reported in previous studies.

Study	Year	No. of Cases	Age/Sex	Predominant Location	Size (mm)	Morphologic Features	Molecular Findings
Hashimoto et al. [1]	2018	18	62–87 years; 11 F/9 M	Sigmoid colon/rectum (90%)	2–20 (median, 5)	Superficial serration with adenomatous glands	*KRAS* mut, 19/20 (95%); *RSPO2/3* fusions and/or overexpression, 18/20 (90%); truncating *APC* mut, 2/20 (10%); *BRAF* mut, 1/20 (5%)
Mizuguchi et al. [15]	2022	46 *	Predominantly 50–80 years	Sigmoid colon/rectum (88%)	3–20 (median, 6)	Sessile or slightly elevated lesions with superficial serration	*KRAS* mut, 96%; *RSPO2/3* fusions and/or overexpression, 92%
Chen et al. [5]	2024	1	68/F	Sigmoid colon	10	Sessile	N/A
Gao et al. [14]	2025	1	38/M	Rectum	21	Slightly elevated	N/A
Present case	2026	1	57/F	Rectum	7	Sessile	*RSPO3* fusion and *KRAS* mut

* Includes isolated and traditional serrated adenoma-associated SuSAs. F: female; M: male; N/A: not applicable; mut: mutations.

## 2. Clinical Summary

A 57-year-old woman with a history of follicular lymphoma, diagnosed approximately 14 years earlier and treated to completion approximately 8 years earlier, presented to our hospital on 3 November, X, with a positive fecal occult blood test. She reported occasional abdominal pain and diarrhea.

Total colonoscopy performed on 27 November, X, revealed mildly decreased vascular visibility and erosions throughout the colon. Mild UC, with a Mayo endoscopic subscore of 1, was suspected, and histopathological examination of biopsy specimens revealed findings consistent with UC.

However, a small erosion was noted at the ileocecal valve, and a biopsy specimen was obtained from this lesion. Colonoscopy revealed erythema and erosions in the cecum (four biopsies taken), ascending colon (two biopsies taken), and rectum (one biopsy taken) (Figure 1a,b). Although the severity was mild, the inflammation was continuous. We identified a 7 mm pale, sessile polyp with a smooth surface in the rectum (Figure 1c). Histopathological examination of the biopsy specimens was consistent with resolving-phase UC. These findings led to a new diagnosis of UC. In addition, histological evaluation of the 7 mm polyp indicated SuSA, for which cold snare polypectomy was successfully performed. Treatment for the UC consisted of 5-aminosalicylic acid 3600 mg/day. Her abdominal pain and diarrhea gradually improved. The patient was followed up with clinical examinations, colonoscopy, and histology. On repeat total colonoscopy performed on May 28 (X + 3), the colonic mucosal lesions had also improved, with a Mayo endoscopic subscore of 0. No recurrence of serrated lesions was observed during the 29-month follow-up period.

## 3. Pathological Findings

### 3.1. Histopathologic Findings

The background colorectal mucosa showed features of resolving-phase UC, including crypt distortion, reduced crypt density, and focal cryptitis (Figure 2a,c). However, basal plasmacytosis and diffuse inflammatory-cell infiltration in the stroma were not identified (Figure 2a). The stroma showed increased eosinophils (Figure 2b). No epithelioid granulomas or infectious organisms were observed.

The SuSA arose in the rectal mucosa and was associated with mild chronic active colitis. No adjacent dysplasia was identified in the surrounding mucosa. Histologically, the lesion showed a characteristic hybrid architecture consisting of tubular adenoma-like deep glands and superficial serration (Figure 2d). Serration was restricted to the superficial portion of the glands, involving the upper one-third to one-half of the crypts. In contrast, the deep crypt bases lacked serration and retained a tubular adenoma-like configuration (Figure 2e,f). The proliferative zone was located predominantly in the basal-to-middle portions of the crypts. No ectopic crypt formation was identified.

The lesion did not show basal crypt dilation or the L-/T-shaped crypt architecture characteristic of an SSL, nor did it show ectopic crypt formation and characteristic cytologic features of a TSA. The combination of superficial serration, adenoma-like deep glands, and a basal-to-middle proliferative zone supported the diagnosis of SuSA. The polypectomy specimen showed complete excision of the lesion, and the resection margins were assessable.

### 3.2. Genetic Findings

For fusion gene analysis, we extracted total RNA from formalin-fixed, paraffin-embedded tissue and performed reverse transcriptase–polymerase chain reaction (RT-PCR) using primers designed based on previously reported *PTPRK::RSPO3* fusion sequences [8].

RT-PCR followed by Sanger sequencing confirmed the *PTPRK::RSPO3* fusion transcripts (Figure 3). These findings are most consistent with alternative transcripts arising from the same fusion event, although independent fusion events cannot be entirely excluded. The fusion transcripts were not independently examined in spatially separate areas of the polyp or in the underlying non-neoplastic mucosa.

Meanwhile, the only variant classified as pathogenic in ClinVar was *KRAS* Q61H. The *KRAS* Q61H mutation had a variant allele frequency of 30.2% and a read depth of 2597×. The lesional area was identified on the corresponding hematoxylin and eosin-stained section, and the formalin-fixed paraffin-embedded block was manually trimmed to enrich the lesional component. The estimated lesional cellularity after macrodissection was approximately 50–60%. Although the observed variant allele frequency was consistent with this estimate, this single-region analysis could not determine clonality or intralesional heterogeneity. The variant passed the sequencing quality filters with a quality score of 100. In addition to *KRAS,* several other variants were detected by the targeted sequencing panel. These additional variants are summarized in Supplementary Table S1.

## 4. Discussion

The present lesion was morphologically consistent with SuSA, based on the characteristic combination of superficial serration, adenoma-like deep glands, and a basal-to-middle proliferative zone. Serration was confined to the upper one-third to one-half of the crypts, whereas the deep crypt bases lacked serration and retained a tubular adenoma-like architecture. Ectopic crypt formation was not identified, and the absence of basal crypt dilation or L-/T-shaped crypt architecture argued against TSA and SSL, respectively [1,6,8,9]. The surrounding mucosa showed resolving-phase UC, characterized by crypt distortion, reduced crypt density, and focal cryptitis, but lacked basal plasmacytosis and diffuse stromal inflammatory-cell infiltration. The lesion itself showed only mild chronic active inflammation, and no adjacent dysplasia was identified. Thus, the vertical distribution of serration and the architecture of the deep crypts, together with the relatively limited inflammatory changes in the surrounding mucosa, supported the diagnosis of SuSA rather than an IBD-associated serrated dysplastic lesion.

The molecular findings further supported the classification of the lesion as SuSA. Targeted sequencing identified a *KRAS* p.Q61H mutation, and RT-PCR followed by Sanger sequencing demonstrated a *PTPRK::RSPO3* fusion. SuSAs have been reported to harbor alterations involving *KRAS* and *RSPO* genes, consistent with activation of the MAPK and WNT signaling pathways [1,8,9]. Both *KRAS* mutations and *RSPO3* fusions have been described in sporadic SuSA [1,8,9,16], and the coexistence of these alterations in the present lesion provides additional molecular support for its classification as SuSA. However, because we analyzed a single macrodissected region, we could not assess the clonality and spatial distribution of these alterations. We also did not perform immunohistochemical analyses of MLH1, MSH2, and p53, which is an additional limitation of this case. Nevertheless, the concordance between the characteristic morphology and the molecular profile supports the interpretation that this lesion belongs to the SuSA spectrum.

Distinguishing sporadic SuSA from IBD-associated non-conventional serrated dysplasia (NCSD) can be challenging because both may exhibit serrated epithelial architecture [10,12,15,17]. IBD-associated NCSD encompasses lesions that may resemble SSL, TSA, or other serrated lesions, and diagnosis requires integrating morphology, clinical background, and, when available, molecular findings [12,15,17,18,19,20]. In the present case, the characteristic superficial serration and adenoma-like deep glands, absence of ectopic crypt formation and SSL-type basal crypt distortion, limited perilesional inflammation, absence of adjacent dysplasia, and the presence of *KRAS* mutation and *PTPRK::RSPO3* fusion collectively favored sporadic SuSA over IBD-associated NCSD. Importantly, the patient had a relatively short history of UC (approximately 30 months), and the disease was resolving when the lesion was detected. Although the lesion arose in colonic mucosa affected by UC, these findings do not establish a causal relationship between chronic inflammation and SuSA development. Rather, this observation is compatible with sporadic SuSA in a patient with UC. However, a single case cannot exclude the influence of the inflammatory microenvironment.

This case also contributes to the limited literature on SuSA in patients with UC. Gao et al. reported a UC-associated SuSA arising in the rectum, measuring approximately 21 mm, with no recurrence during 11 months of follow-up; however, they did not perform molecular analysis [14]. Molecular studies of sporadic SuSAs have demonstrated frequent alterations involving *KRAS* and *RSPO*. Hashimoto et al. identified *KRAS* mutations in 19 of 20 cases (95%) and *RSPO* fusions and/or overexpression in 18 cases (90%) [1]. In contrast, Mizuguchi et al. reported *KRAS* mutations in 96% and *RSPO* fusions/overexpression in 92% of molecularly characterized SuSAs [15]. These findings indicate that *KRAS* and *RSPO* alterations are characteristic molecular features of sporadic SuSAs. The present case expands the molecular spectrum of SuSA by demonstrating a *PTPRK::RSPO3* fusion together with a *KRAS* Q61H mutation in a patient with UC. Nevertheless, the single-case design, single-institution setting, relatively short UC duration, and lack of population-based data on SuSA incidence in Japanese patients limit interpretation. The absence of MLH1, MSH2, and p53 immunohistochemistry and the inability to assess the clonality or spatial distribution of the molecular alterations are additional limitations. The previous lymphoma treatment and immunosuppressive therapies may also have affected the intestinal microbiota or local mucosal immunity, although their contribution to the present lesion cannot be determined. Larger multicenter clinicopathological and molecular studies are needed to clarify whether SuSA in patients with UC represents an incidental sporadic lesion or a distinct subset associated with the inflammatory microenvironment.

## 5. Conclusions

In conclusion, we report a solitary SuSA arising in a patient with UC. The lesion exhibited histological and molecular features consistent with sporadic SuSA. Although the lesion developed in colonic mucosa affected by UC, the short duration of UC and mild, resolving inflammation do not support a causal relationship between UC and SuSA development. The present case therefore represents an isolated occurrence of SuSA in a patient with UC and does not provide evidence that UC increases the risk of SuSA. Further population-based and multicenter clinicopathological and molecular studies are required to determine whether SuSA occurs more frequently in patients with UC than in the general population.

## Figures and Tables

**Figure 1:**
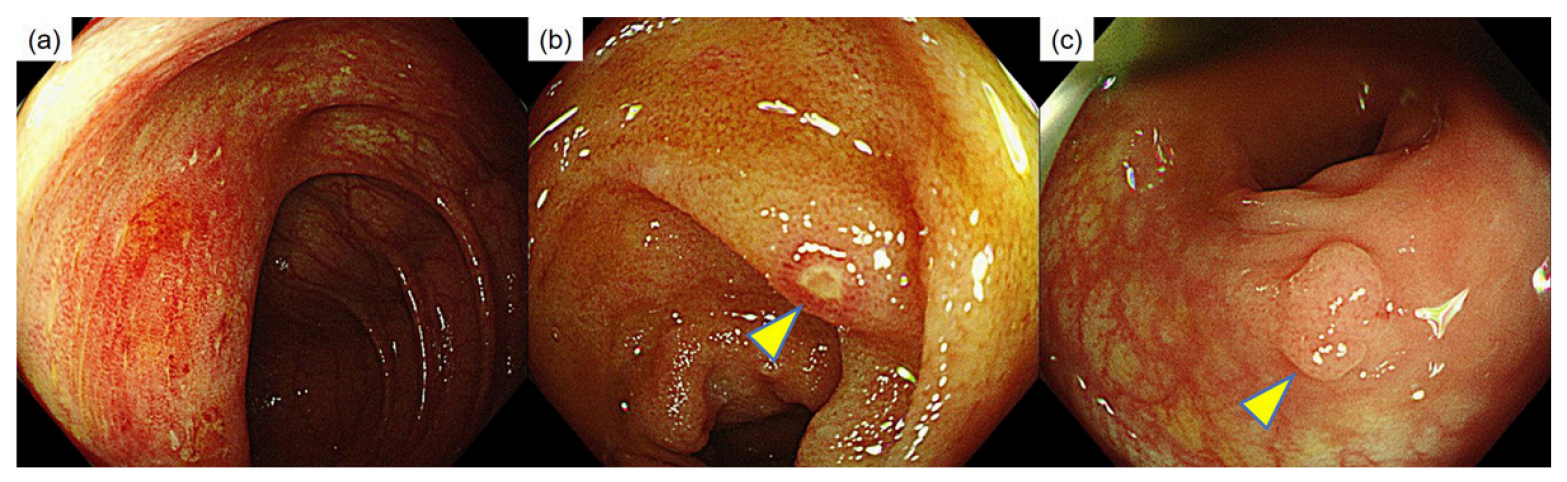
Endoscopic findings. (**a**) The cecal mucosa shows diffuse erythema with loss of the normal vascular pattern. (**b**) Multiple small, round erosions are scattered at the ileocecal valve (arrowhead). (**c**) A pale, sessile polyp with a smooth surface, measuring 7 mm in diameter, is identified in the rectum (arrowhead).

**Figure 2:**
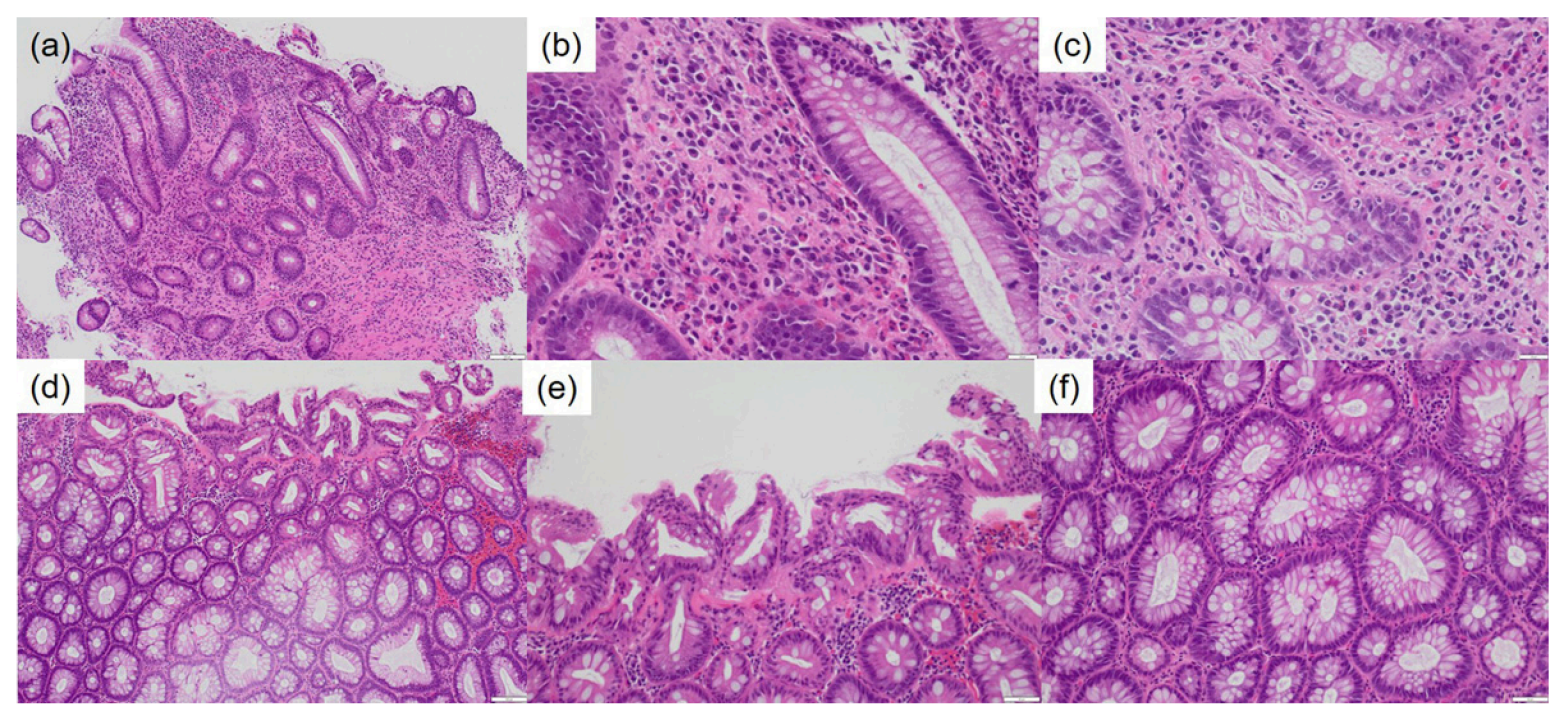
Histopathological findings of the colorectal mucosa in the background biopsy. (**a**) Histological appearance of resolving-stage UC. The biopsy showed crypt distortion and reduced crypt density; however, basal plasmacytosis and diffuse inflammatory-cell infiltration in the stroma were not identified (bar = 100 μm). (**b**) Eosinophils increased in the stroma (bar = 20 μm). (**c**) Some crypts showed cryptitis (bar = 20 μm). (**d**) Histopathological findings of the rectal SuSA (bar = 100 μm). (**e**) Serration is confined to the upper one-third to one-half of the glands, whereas the deep crypt bases lack it (bar = 50 μm). (**f**) The proliferative zone is located in the basal to middle portions of the crypts, and the overall glandular architecture resembles that of a low-grade tubular adenoma (bar = 50 μm).

**Figure 3:**
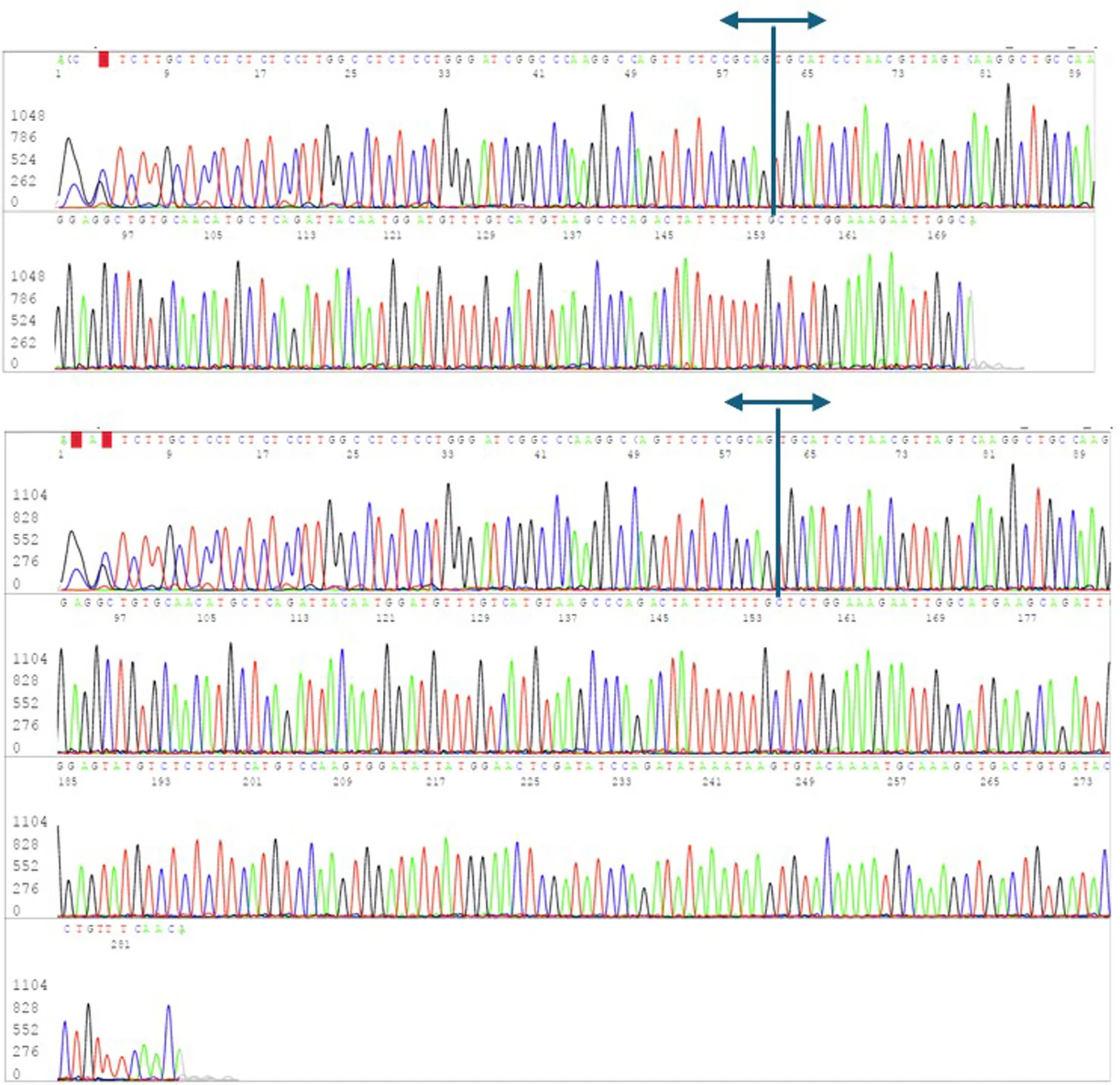
Molecular findings. RT-PCR followed by Sanger sequencing confirmed *PTPRK::RSPO3* fusion transcripts.

## Data Availability

The original contributions presented in this study are included in the article/Supplementary Materials. Further inquiries can be directed to the corresponding author.

## Supplementary Materials

The following supporting information can be downloaded at: https://www.mdpi.com/article/10.3390/diagnostics16193147/s1, Table S1: Detailed clinicopathological and molecular features of superficially serrated adenomas reported in previous studies.

## Abbreviations

The following abbreviations are used in this manuscript:

IBD	Inflammatory bowel disease
NCSD	Non-conventional serrated dysplasia
RT-PCR	Reverse transcriptase–polymerase chain reaction.
SSL	Sessile serrated lesion
SuSA	Superficially serrated adenoma
TSA	Traditional serrated adenoma
UC	Ulcerative colitis
