# Supplementary material for: Isolated Superficially Serrated Adenoma in Ulcerative Colitis: A Case Report with Molecular Analysis in Japan
Source: Diagnostics (Basel). 2026 Sep 28;16(19):3147. doi: 10.3390/diagnostics16193147 (PMC13664024; doi:10.3390/diagnostics16193147)
Supplement: Supplementary file 1 [file diagnostics-16-03147-s001.zip › diagnostics-4537757-supplementary.pdf]

Supplemental Table S1. Detailed clinicopathological and molecular features of superficially serrated adenomas reported in previous studies.

Author	Year	Age/Sex	Location	Size	Morphology	Molecular findings			Follow up
				(mm)		*	**	***	
Hashimoto [1]	2018	62/F	Rectum	5	N/A	*RSPO3* fusion	*KRAS* mut	Negative	N/A
		68/F	Rectum	15	N/A	*RSPO3* fusion	*KRAS* mut	Negative	N/A
		77/F	Sigmoid	20	N/A	*RSPO3* overexpression	*KRAS* mut	Negative	N/A
		63/F	Rectum	10	N/A	*RSPO3* overexpression	*KRAS* mut	Negative	N/A
		72/F	Sigmoid	5	N/A	*RSPO3* fusion	*KRAS* mut	Negative	N/A
		62/M	Sigmoid	18	N/A	*RSPO2* overexpression	*KRAS* mut	Negative	N/A
		66/M	Rectum	5	N/A	*RSPO3* fusion	*BRAF* mut	Negative	N/A
		78/F	Transverse	3	N/A	*RSPO3* fusion	*KRAS* mut	Negative	N/A
		79/M	Sigmoid	4	N/A	*RSPO3* overexpression	*KRAS* mut	Negative	N/A
		69/M	Rectum	9	N/A	*APC* mut	*KRAS* mut	*GNAS* mut	N/A
		62/F	Ascending	2	N/A	*RSPO3* overexpression	*KRAS* mut	Negative	N/A
		71/M	Sigmoid	5	N/A	*RSPO3* fusion	*KRAS* mut	Negative	N/A
		65/F	Rectum	9	N/A	*RSPO3* fusion	*KRAS* mut	Negative	N/A
		87/M	Rectum	5	N/A	*RSPO3* fusion	*KRAS* mut	Negative	N/A
		83/M	Sigmoid	2	N/A	*RSPO3* fusion	*KRAS* mut	Negative	N/A
		76/M	Rectum	5	N/A	*RSPO3* overexpression	*KRAS* mut	Negative	N/A
		71/F	Rectum	4	N/A	*RSPO3* fusion	*KRAS* mut	Negative	N/A
		64/F	Rectum	4	N/A	*RSPO2* overexpression	*KRAS* mut	Negative	N/A
		72/M	Rectum	3	N/A	*RSPO3* fusion	*KRAS* mut	Negative	N/A

		70/F	Sigmoid	3	N/A	*APC* mut	*KRAS* mut	Negative	N/A
Mizuguchi [15]	2022	62/F	Rectum	7	Slightly elevated	*RSPO3* fusion	*KRAS* mut	Negative	N/A
		68/F	Rectum	15	Slightly elevated	*RSPO3* fusion	*KRAS* mut	Negative	N/A
		77/F	Sigmoid	20	Slightly elevated	*RSPO3* overexpression	*KRAS* mut	Negative	N/A
		63/F	Rectum	10	Slightly elevated	*RSPO3* overexpression	*KRAS* mut	Negative	N/A
		72/F	Sigmoid	6	Slightly elevated	*RSPO3* fusion	*KRAS* mut	Negative	N/A
		62/M	Sigmoid	20	Slightly elevated	*RSPO2* overexpression	*KRAS* mut	Negative	N/A
		66/M	Rectum	5	Slightly elevated	*RSPO3* fusion	*BRAF* mut	Negative	N/A
		78/F	Transverse	6	Slightly elevated	*RSPO3* fusion	*KRAS* mut	Negative	N/A
		79/M	Sigmoid	4	Slightly elevated	*RSPO3* overexpression	*KRAS* mut	Negative	N/A
		69/M	Rectum	9	Slightly elevated	*APC* mut	*KRAS* mut	*GNAS* mut	N/A
		62/F	Ascending	3	Slightly elevated	*RSPO3* overexpression	*KRAS* mut	Negative	N/A
		65/F	Rectum	9	Sessile	*RSPO3* fusion	*KRAS* mut	Negative	N/A
		87/M	Rectum	8	Sessile	*RSPO3* fusion	*KRAS* mut	Negative	N/A
		76/M	Rectum	6	Sessile	*RSPO3* overexpression	*KRAS* mut	Negative	N/A
		71/F	Rectum	5	Sessile	*RSPO3* fusion	*KRAS* mut	Negative	N/A
		64/F	Rectum	6	Slightly elevated	*RSPO2* overexpression	*KRAS* mut	Negative	N/A
		72/M	Rectum	3	Slightly elevated	*RSPO3* fusion	*KRAS* mut	Negative	N/A
		70/F	Sigmoid	5	Sessile	*APC* mut	*KRAS* mut	Negative	N/A
		71/F	Rectum	9	Slightly elevated	*RSPO3* fusion	*KRAS* mut	Negative	N/A
		69/F	Descending	10	Slightly elevated	*RSPO3* fusion	*KRAS* mut	Negative	N/A
		54/M	Rectum	4	Sessile	*RSPO3* fusion	*KRAS* mut	Negative	N/A
		74/M	Sigmoid	12	Slightly elevated	*RSPO3* fusion	*KRAS* mut	Negative	N/A

		71/M	Sigmoid	18	Sessile	*RSPO3* fusion	*KRAS* mut	Negative	N/A
		80/F	Rectum	5	Sessile	*RSPO2* overexpression	*KRAS* mut	Negative	N/A
		74/F	Sigmoid	5	Sessile	*RSPO3* fusion	*KRAS* mut	Negative	N/A
Chen [5]	2024	68/F	Sigmoid	10	Sessile	N/A	N/A	N/A	N/A
Gao [14]	2025	38/M	Rectum	21	Slightly elevated	N/A	N/A	N/A	11 months<br>No recurrence
Present case	2026	57/F	Rectum	7	Sessile	*RSPO3* fusion	*KRAS* mut	Negative	29 months<br>No recurrence

*WNT pathway gene alterations; **KRAS/BRAF mutations; ***GNAS mutations; F: female; M: male; N/A: not applicable; mut: mutation
